# Report on computational assessment of Tumor Infiltrating Lymphocytes from the International Immuno-Oncology Biomarker Working Group

**DOI:** 10.1038/s41523-020-0154-2

**Published:** 2020-05-12

**Authors:** Mohamed Amgad, Elisabeth Specht Stovgaard, Eva Balslev, Jeppe Thagaard, Weijie Chen, Sarah Dudgeon, Ashish Sharma, Jennifer K. Kerner, Carsten Denkert, Yinyin Yuan, Khalid AbdulJabbar, Stephan Wienert, Peter Savas, Leonie Voorwerk, Andrew H. Beck, Anant Madabhushi, Johan Hartman, Manu M. Sebastian, Hugo M. Horlings, Jan Hudeček, Francesco Ciompi, David A. Moore, Rajendra Singh, Elvire Roblin, Marcelo Luiz Balancin, Marie-Christine Mathieu, Jochen K. Lennerz, Pawan Kirtani, I-Chun Chen, Jeremy P. Braybrooke, Giancarlo Pruneri, Sandra Demaria, Sylvia Adams, Stuart J. Schnitt, Sunil R. Lakhani, Federico Rojo, Laura Comerma, Sunil S. Badve, Mehrnoush Khojasteh, W. Fraser Symmans, Christos Sotiriou, Paula Gonzalez-Ericsson, Katherine L. Pogue-Geile, Rim S. Kim, David L. Rimm, Giuseppe Viale, Stephen M. Hewitt, John M. S. Bartlett, Frédérique Penault-Llorca, Shom Goel, Huang-Chun Lien, Sibylle Loibl, Zuzana Kos, Sherene Loi, Matthew G. Hanna, Stefan Michiels, Marleen Kok, Torsten O. Nielsen, Alexander J. Lazar, Zsuzsanna Bago-Horvath, Loes F. S. Kooreman, Jeroen A. W. M. van der Laak, Joel Saltz, Brandon D. Gallas, Uday Kurkure, Michael Barnes, Roberto Salgado, Lee A. D. Cooper, International Immuno-Oncology Biomarker Working Group, Aini Hyytiäinen, Akira I. Hida, Alastair Thompson, Alex Lefevre, Allen Gown, Amy Lo, Anna Sapino, Andre Moreira, Andrea Richardson, Andrea Vingiani, Andrew M. Bellizzi, Andrew Tutt, Angel Guerrero-Zotano, Anita Grigoriadis, Anna Ehinger, Anna C. Garrido-Castro, Anne Vincent-Salomon, Anne-Vibeke Laenkholm, Ashley Cimino-Mathews, Ashok Srinivasan, Balazs Acs, Baljit Singh, Benjamin Calhoun, Benjamin Haibe-Kans, Benjamin Solomon, Bibhusal Thapa, Brad H. Nelson, Carlos Castaneda, Carmen Ballesteroes-Merino, Carmen Criscitiello, Carolien Boeckx, Cecile Colpaert, Cecily Quinn, Chakra S. Chennubhotla, Charles Swanton, Cinzia Solinas, Crispin Hiley, Damien Drubay, Daniel Bethmann, Deborah A. Dillon, Denis Larsimont, Dhanusha Sabanathan, Dieter Peeters, Dimitrios Zardavas, Doris Höflmayer, Douglas B. Johnson, E. Aubrey Thompson, Edi Brogi, Edith Perez, Ehab A. ElGabry, Elizabeth F. Blackley, Emily Reisenbichler, Enrique Bellolio, Ewa Chmielik, Fabien Gaire, Fabrice Andre, Fang-I Lu, Farid Azmoudeh-Ardalan, Forbius Tina Gruosso, Franklin Peale, Fred R. Hirsch, Frederick Klaushen, Gabriela Acosta-Haab, Gelareh Farshid, Gert van den Eynden, Giuseppe Curigliano, Giuseppe Floris, Glenn Broeckx, Harmut Koeppen, Harry R. Haynes, Heather McArthur, Heikki Joensuu, Helena Olofsson, Ian Cree, Iris Nederlof, Isabel Frahm, Iva Brcic, Jack Chan, Jacqueline A. Hall, James Ziai, Jane Brock, Jelle Wesseling, Jennifer Giltnane, Jerome Lemonnier, Jiping Zha, Joana M. Ribeiro, Jodi M. Carter, Johannes Hainfellner, John Le Quesne, Jonathan W. Juco, Jorge Reis-Filho, Jose van den Berg, Joselyn Sanchez, Joseph Sparano, Joël Cucherousset, Juan Carlos Araya, Julien Adam, Justin M. Balko, Kai Saeger, Kalliopi Siziopikou, Karen Willard-Gallo, Karolina Sikorska, Karsten Weber, Keith E. Steele, Kenneth Emancipator, Khalid El Bairi, Kim R. M. Blenman, Kimberly H. Allison, Koen K. van de Vijver, Konstanty Korski, Lajos Pusztai, Laurence Buisseret, Leming Shi, Liu Shi-wei, Luciana Molinero, M. Valeria Estrada, Maartje van Seijen, Magali Lacroix-Triki, Maggie C. U. Cheang, Maise al Bakir, Marc van de Vijver, Maria Vittoria Dieci, Marlon C. Rebelatto, Martine Piccart, Matthew P. Goetz, Matthias Preusser, Melinda E. Sanders, Meredith M. Regan, Michael Christie, Michael Misialek, Michail Ignatiadis, Michiel de Maaker, Mieke van Bockstal, Miluska Castillo, Nadia Harbeck, Nadine Tung, Nele Laudus, Nicolas Sirtaine, Nicole Burchardi, Nils Ternes, Nina Radosevic-Robin, Oleg Gluz, Oliver Grimm, Paolo Nuciforo, Paul Jank, Petar Jelinic, Peter H. Watson, Prudence A. Francis, Prudence A. Russell, Robert H. Pierce, Robert Hills, Roberto Leon-Ferre, Roland de Wind, Ruohong Shui, Sabine Declercq, Sam Leung, Sami Tabbarah, Sandra C. Souza, Sandra O’Toole, Sandra Swain, Scooter Willis, Scott Ely, Seong- Rim Kim, Shahinaz Bedri, Sheeba Irshad, Shi-Wei Liu, Shona Hendry, Simonetta Bianchi, Sofia Bragança, Soonmyung Paik, Stephen B. Fox, Stephen J. Luen, Stephen Naber, Sua Luz, Susan Fineberg, Teresa Soler, Thomas Gevaert, Timothy d’Alfons, Tom John, Tomohagu Sugie, Veerle Bossuyt, Venkata Manem, Vincente Peg Cámaea, Weida Tong, Wentao Yang, William T. Tran, Yihong Wang, Yves Allory, Zaheed Husain

**Affiliations:** 1 Department of Biomedical Informatics, Emory University School of Medicine, Atlanta, GA USA; 2 Department of Pathology, Herlev and Gentofte Hospital, University of Copenhagen, Herlev, Denmark; 3 DTU Compute, Department of Applied Mathematics, Technical University of Denmark, Lyngby, Denmark; 4 Visiopharm A/S, Hørsholm, Denmark; 5 FDA/CDRH/OSEL/Division of Imaging, Diagnostics, and Software Reliability, Silver Spring, MD USA; 6 PathAI, Cambridge, MA USA; 7 Institut für Pathologie, Universitätsklinikum Gießen und Marburg GmbH, Standort Marburg, Philipps-Universität Marburg, Marburg, Germany; 8 Institute of Pathology, Philipps-University Marburg, Marburg, Germany; 9 German Cancer Consortium (DKTK), Partner Site Charité, Berlin, Germany; 10 Centre for Evolution and Cancer, The Institute of Cancer Research, London, UK; 11 Division of Molecular Pathology, The Institute of Cancer Research, London, UK; 12 Division of Research and Cancer Medicine, Peter MacCallum Cancer Centre, University of Melbourne, Victoria, Australia; 13 Sir Peter MacCallum Department of Oncology, University of Melbourne, Parkville, Australia; 14 Department of Tumor Biology & Immunology, The Netherlands Cancer Institute, Amsterdam, The Netherlands; 15 Case Western Reserve University, Department of Biomedical Engineering, Cleveland, OH USA; 16 Louis Stokes Cleveland Veterans Administration Medical Center, Cleveland, OH USA; 17 Department of Oncology and Pathology, Karolinska Institutet and University Hospital, Solna, Sweden; 18 Departments of Epigenetics and Molecular Carcinogenesis, The University of Texas MD Anderson Cancer Center, Houston, TX USA; 19 Division of Molecular Pathology, The Netherlands Cancer Institute, Amsterdam, The Netherlands; 20 Department of Research IT, The Netherlands Cancer Institute, Amsterdam, The Netherlands; 21 Department of Pathology, Radboud University Medical Center, Nijmegen, The Netherlands; 22 Department of Pathology, UCL Cancer Institute, London, UK; 23 Department of Pathology and Laboratory Medicine, Icahn School of Medicine at Mount Sinai, New York, NY USA; 24 Université Paris-Saclay, Univ. Paris-Sud, Villejuif, France; 25 Department of Pathology, Faculty of Medicine, University of São Paulo, São Paulo, Brazil; 26 Department of Medical Biology and Pathology, Gustave Roussy Cancer Campus, Villejuif, France; 27 Department of Pathology, Massachusetts General Hospital, Boston, MA USA; 28 Department of Histopathology, Manipal Hospitals Dwarka, New Delhi, India; 29 Department of Oncology, National Taiwan University Cancer Center, Taipei, Taiwan; 30 Nuffield Department of Population Health, University of Oxford, Oxford, UK; 31 Department of Medical Oncology, University Hospitals Bristol NHS Foundation Trust, Bristol, UK; 32 Pathology Department, Fondazione IRCCS Istituto Nazionale Tumori and University of Milan, School of Medicine, Milan, Italy; 33 Weill Cornell Medical College, New York, NY USA; 34 Laura and Isaac Perlmutter Cancer Center, NYU Langone Medical Center, New York, NY USA; 35 Department of Pathology, Brigham and Women’s Hospital, Boston, MA USA; 36 The University of Queensland Centre for Clinical Research and Pathology Queensland, Brisbane, Australia; 37 Pathology Department, CIBERONC-Instituto de Investigación Sanitaria Fundación Jiménez Díaz (IIS-FJD), Madrid, Spain; 38 GEICAM-Spanish Breast Cancer Research Group, Madrid, Spain; 39 Department of Pathology and Laboratory Medicine, Indiana University School of Medicine, Indianapolis, IN USA; 40 Roche Tissue Diagnostics, Digital Pathology, Santa Clara, CA USA; 41 Department of Pathology, The University of Texas MD Anderson Cancer Center, Houston, TX USA; 42 Breast Cancer Translational Research Laboratory, Institut Jules Bordet, Université Libre de Bruxelles (ULB), Brussels, Belgium; 43 ULB-Cancer Research Center (U-CRC) Université Libre de Bruxelles, Brussels, Belgium; 44 Breast Cancer Program, Vanderbilt-Ingram Cancer Center, Vanderbilt University Medical Center, Nashville, TN USA; 45 NRG Oncology/NSABP, Pittsburgh, PA USA; 46 Department of Pathology, Yale University School of Medicine, New Haven, CT USA; 47 Department of Pathology, IEO, European Institute of Oncology IRCCS & State University of Milan, Milan, Italy; 48 Laboratory of Pathology, National Cancer Institute, National Institutes of Health, Bethesda, MD USA; 49 Ontario Institute for Cancer Research, Toronto, ON Canada; 50 Edinburgh Cancer Research Centre, Western General Hospital, Edinburgh, UK; 51 Department of Pathology and Molecular Pathology, Centre Jean Perrin, Clermont-Ferrand, France; 52 UMR INSERM 1240, Universite Clermont Auvergne, Clermont-Ferrand, France; 53 Victorian Comprehensive Cancer Centre building, Peter MacCallum Cancer Centre, Melbourne, Victoria Australia; 54 Department of Pathology, National Taiwan University Hospital, Taipei, Taiwan; 55 German Breast Group, c/o GBG-Forschungs GmbH, Neu-Isenburg, Germany; 56 Department of Pathology, BC Cancer, Vancouver, British Columbia Canada; 57 Peter MacCallum Cancer Centre, Melbourne, Australia; 58 Department of Pathology, Memorial Sloan Kettering Cancer Center, New York, NY USA; 59 Gustave Roussy, Universite Paris-Saclay, Villejuif, France; 60 Université Paris-Sud, Institut National de la Santé et de la Recherche Médicale, Villejuif, France; 61 Division of Molecular Oncology & Immunology, The Netherlands Cancer Institute, Amsterdam, The Netherlands; 62 Department of Medical Oncology, The Netherlands Cancer Institute, Amsterdam, The Netherlands; 63 University of British Columbia, Vancouver, British Columbia Canada; 64 Department of Genomic Medicine, The University of Texas MD Anderson Cancer Center, Houston, TX USA; 65 Department of Translational Molecular Pathology, The University of Texas MD Anderson Cancer Center, Houston, TX USA; 66 Department of Dermatology, The University of Texas MD Anderson Cancer Center, Houston, TX USA; 67 Department of Pathology, Medical University of Vienna, Vienna, Austria; 68 GROW - School for Oncology and Developmental Biology, Maastricht University Medical Centre, Maastricht, The Netherlands; 69 Department of Pathology, Maastricht University Medical Centre, Maastricht, The Netherlands; 70 Center for Medical Image Science and Visualization, Linköping University, Linköping, Sweden; 71 Department of Biomedical Informatics, Stony Brook University, Stony Brook, NY USA; 72 Roche Diagnostics Information Solutions, Belmont, CA USA; 73 Department of Pathology, GZA-ZNA Ziekenhuizen, Antwerp, Belgium; 74 Department of Pathology, Northwestern University Feinberg School of Medicine, Chicago, IL USA; 75 Department of Oral and Maxillofacial Diseases, Helsinki, Finland; 76 Department of Pathology, Matsuyama Shimin Hospital, Matsuyama, Japan; 77 Surgical Oncology, Baylor College of Medicine, Texas, USA; 78 Roche Diagnostics, Machelen, Belgium; 79 PhenoPath Laboratories, Seattle, USA; 80 Research Pathology, Genentech Inc., South San Francisco, USA; 81 Department of Medical Sciences, University of Turin, Italy and Candiolo Cancer Institute - FPO, IRCCS, Candiolo, Italy; 82 Pulmonary Pathology, New York University Center for Biospecimen Research and Development, New York University, New York, NY USA; 83 Department of Pathology, Johns Hopkins Hospital, Baltimore, USA; 84 Department of Pathology, Istituto Europeo di Oncologia, University of Milan, Milan, Italy; 85 Department of Pathology, University of Iowa Hospitals and Clinics, Iowa City, USA; 86 Breast Cancer Now Toby Robins Research Centre, The Institute of Cancer Research, London, UK; 87 Department of Oncology, Instituto Valenciano de Oncología, Valencia, Spain; 88 Cancer Bioinformatics Lab, Cancer Centre at Guy’s Hospital, London, UK; 89 School of Life Sciences and Medicine, King’s College London, London, UK; 90 Department of Clinical Genetics and Pathology, Skåne University Hospital, Lund University, Lund, Sweden; 91 Dana-Farber Cancer Institute, Boston, MA USA; 92 Institut Curie, Paris Sciences Lettres Université, Inserm U934, Department of Pathology, Paris, France; 93 Department of Surgical Pathology Zealand University Hospital, Køge, Denmark; 94 Departments of Pathology and Oncology, The Johns Hopkins Hospital, Baltimore, USA; 95 National Surgical Adjuvant Breast and Bowel Project Operations Center/NRG Oncology, Pittsburgh, PA USA; 96 Department of Pathology, Karolinska Institute, Solna, Sweden; 97 Department of Pathology, New York University Langone Medical Centre, New York, USA; 98 Department of Pathology and Laboratory Medicine, UNC School of Medicine, Columbia, USA; 99 Québec Heart and Lung Institute Research Center, Laval University, Quebec city, Quebec Canada; 100 Department of Medical Oncology, Peter MacCallum Cancer Centre, Melbourne, VIC Australia; 101 Department of Medicine, University of Melbourne, Parkville, Australia; 102 Trev & Joyce Deeley Research Centre, British Columbia Cancer Agency, Victoria, Canada; 103 Department of Medical Oncology, Instituto Nacional de Enfermedades Neoplásicas, Lima, Perú; 104 Department of Research, Instituto Nacional de Enfermedades Neoplasicas, Lima, 15038 Peru; 105 Providence Cancer Research Center, Portland, Oregon, USA; 106 Department of Medical Oncology, Istituto Europeo di Oncologia, Milan, Italy; 107 Department of Pathology, AZ Turnhout, Turnhout, Belgium; 108 Department of Pathology, St Vincent’s University Hospital and University College Dublin, Dublin, Ireland; 109 Department of Computational and Systems Biology, University of Pittsburgh, Pittsburgh, USA; 110 Cancer Research UK Lung Cancer Centre of Excellence, University College London Cancer Institute, University College London, London, UK; 111 Azienda AUSL, Regional Hospital of Aosta, Aosta, Italy; 112 University Hospital Halle (Saale), Institute of Pathology, Halle, Saale Germany; 113 Department of Pathology, Brigham and Women’s Hospital, Boston, MA Department of Pathology, Dana Farber Cancer Institute, Boston, MA USA; 114 Department of Pathology, Jules Bordet Institute, Brussels, Belgium; 115 Department of Clinical Medicine, Macquarie University, Sydney, Australia; 116 HistoGeneX NV, Antwerp, Belgium and AZ Sint-Maarten Hospital, Mechelen, Belgium; 117 Oncology Clinical Development, Bristol-Myers Squibb, Princeton, USA; 118 Institut für Pathologie, UK Hamburg, Hamburg, Germany; 119 Department of Medicine, Vanderbilt University Medical Centre, Nashville, USA; 120 Department of Cancer Biology, Mayo Clinic, Jacksonville, USA; 121 Department of Oncology, Mayo Clinic, Rochester, USA; 122 Roche, Tucson, USA; 123 Department of Pathology, Universidad de La Frontera, Temuco, Chile; 124 Departamento de Anatomía Patológica, Universidad de La Frontera, Temuco, Chile; 125 Tumor Pathology Department, Maria Sklodowska-Curie Memorial Cancer Center, Gliwice, Poland; 126 Pathology and Tissue Analytics, Roche, Machelen, Belgium; 127 Department of Medical Oncology, Gustave Roussy, Villejuif, France; 128 Sunnybrook Health Sciences Centre, Toronto, Canada; 129 Tehran University of Medical Sciences, Tehran, Iran; 130 Translational Research, Montreal, Canada; 131 Oncology Biomarker Development, Genentech-Roche, Machelen, Belgium; 132 Division of Medical Oncology, Department of Medicine, University of Colorado Anschutz Medical Campus, Aurora, USA; 133 Institute of Pathology, Charité Universitätsmedizin Berlin, Berlin, Germany; 134 Department of Pathology, Hospital de Oncología Maria Curie, Buenos Aires, Argentina; 135 Directorate of Surgical Pathology, SA Pathology, Adelaide, Australia; 136 Department of Pathology, GZA-ZNA Hospitals, Wilrijk, Belgium; 137 University of Milano, Istituto Europeo di Oncologia, IRCCS, Milano, Italy; 138 Division of Early Drug Development for Innovative Therapy, IEO, European Institute of Oncology IRCCS, Milan, Italy; 139 Department of Imaging and Pathology, Laboratory of Translational Cell & Tissue Research, Leuven, Belgium; 140 KU Leuven- University Hospitals Leuven, Department of Pathology, Leuven, Belgium; 141 Department of Pathology, University Hospital Antwerp, Antwerp, Belgium; 142 Translational Health Sciences, Department of Cellular Pathology, North Bristol NHS Trust, University of Bristol, Bristol, UK; 143 Medical Oncology, Department of Medicine, Cedars-Sinai Medical Center, Los Angeles, USA; 144 Helsinki University Central Hospital, Helsinki, Finland; 145 Department of Clinical Pathology, Akademiska University Hospital, Uppsala, Sweden; 146 International Agency for Research on Cancer (IARC), World Health Organization, Lyon, France; 147 Division of Tumor Biology & Immunology, The Netherlands Cancer Institute, Amsterdam, The Netherlands; 148 Department of Pathology, Sanatorio Mater Dei, Buenos Aires, Argentina; 149 Institute of Pathology, Medical University of Graz, Graz, Austria; 150 Department of Oncology, National Cancer Centre, Singapore, Singapore; 151 Vivactiv Ltd, Bellingdon, Bucks, UK; 152 Department of Pathology, Brigham and Women’s Hospital, Boston, USA; 153 Department of Pathology, Netherlands Cancer Institute, Amsterdam, The Netherlands; 154 R&D UNICANCER, Paris, France; 155 Translational Sciences, MedImmune, Gaithersberg, USA; 156 Breast Unit, Champalimaud Clinical Centre, Lisboa, Portugal; 157 Department of Laboratory Medicine and Pathology, Mayo Clinic, Rochester, USA; 158 Department of Medicine, Clinical Division of Oncology, Comprehensive Cancer Centre Vienna, Medical University of Vienna, Vienna, Austria; 159 Leicester Cancer Research Centre, University of Leicester, Leicester, and MRC Toxicology Unit, University of Cambridge, Cambridge, UK; 160 Merck & Co., Inc, Kenilworth, NJ USA; 161 Human Oncology and Pathogenesis Program, Memorial Sloan Kettering Cancer Center, New York, NY USA; 162 Department of Pathology, The Netherlands Cancer Institute, Amsterdam, The Netherlands; 163 Department of Medicine, Department of Obstetrics & Gynecology and Women’s Health, Albert Einstein Medical Center, Bronx, USA; 164 GHI Le Raincy-Montfermeil, Chelles, Île-de-France, France; 165 Department of Pathology, Gustave Roussy, Grand Paris, France; 166 Departments of Medicine and Cancer Biology, Vanderbilt University Medical Centre, Nashville, USA; 167 Vm Scope, Berlin, Germany; 168 Department of Pathology, Breast Pathology Section, Northwestern University, Chicago, USA; 169 Molecular Immunology Unit, Institut Jules Bordet, Université Libre de Bruxelles, Brussels, Belgium; 170 Department of Biometrics, The Netherlands Cancer Institute, Amsterdam, The Netherlands; 171 German Breast Group, Neu-Isenburg, Germany; 172 Cancer Biomarkers Working Group, Faculty of Medicine and Pharmacy, Université Mohamed Premier, Oujda, Morocco; 173 Yale Cancer Center Genetics, Genomics and Epigenetics Program, Yale School of Medicine, New Haven, CT USA; 174 Pathology Department, Stanford University Medical Centre, Stanford, USA; 175 Department of Pathology, University Hospital Ghent, Ghent, Belgium; 176 Pathology and Tissue Analytics, Roche Innovation Centre Munich, Penzberg, Germany; 177 Center for Pharmacogenomics and Fudan-Zhangjiang, Center for Clinical Genomics School of Life Sciences and Shanghai Cancer Center, Fudan University, Fudan, China; 178 Sichuan Cancer Hospital, Chengdu, China; 179 Biorepository and Tissue Technology Shared Resources, University of California San Diego, San Diego, USA; 180 Division of Molecular Pathology, The Netherlands Cancer Institute, Amsterdam, The Netherlands; 181 Department of Pathology, Gustave Roussy, Villejuif, France; 182 Institute of Cancer Research Clinical Trials and Statistics Unit, The Institute of Cancer Research, Surrey, UK; 183 Department of Pathology, Academic Medical Center, Amsterdam, The Netherlands; 184 Department of Surgery, Oncology and Gastroenterology, University of Padova, Padua, Italy; 185 Institut Jules Bordet, Universite Libre de Bruxelles, Brussels, Belgium; 186 Department of Pathology, Microbiology and Immunology, Vanderbilt University Medical Centre, Nashville, USA; 187 Harvard Medical School, Boston, USA; 188 Division of Biostatistics, Dana-Farber Cancer Institute, Boston, USA; 189 Department of Anatomical Pathology, Royal Melbourne Hospital, Parkville, Australia; 190 Vernon Cancer Center, Newton-Wellesley Hospital, Newton, USA; 191 Department of Medical Oncology, Institut Jules Bordet, Université Libre de Bruxelles, Brussels, Belgium; 192 Department of Pathology, Cliniques universitaires Saint-Luc, Brussels, Belgium; 193 Breast Center, Dept. OB&GYN and CCC (LMU), University of Munich, Munich, Germany; 194 Division of Hematology-Oncology, Beth Israel Deaconess Medical Center, Boston, USA; 195 University of Leuven, Leuven, Belgium; 196 Department of Pathology, Institut Jules Bordet, Université Libre de Bruxelles, Brussels, Belgium; 197 German Breast Group GmbH, Neu-Isenburg, Germany; 198 Service de Biostatistique et d’Epidémiologie, Gustave Roussy, CESP, Université-Paris Sud, Université Paris-Saclay, Villejuif, France; 199 Department of Surgical Pathology and Biopathology, Jean Perrin Comprehensive Cancer Centre, Clermont-Ferrand, France; 200 Johanniter GmbH - Evangelisches Krankenhaus Bethesda Mönchengladbach, West German Study Group, Mönchengladbach, Germany; 201 Molecular Oncology Group, Vall d’Hebron Institute of Oncology, Barcelona, Spain; 202 Department of Pathology, University of Marburg, Marburg, Germany; 203 Department of Pathology and Laboratory Medicine, University of British Columbia, Columbia, USA; 204 Department of Anatomical Pathology, St Vincent’s Hospital Melbourne, Fitzroy, Australia; 205 Cancer Immunotherapy Trials Network, Central Laboratory and Program in Immunology, Fred Hutchinson Cancer Research Center, Seattle, USA; 206 Clinical Trial Service Unit & Epidemiological Studies Unit, University of Oxford, Oxford, UK; 207 Department of Pathology, Fudan University Shanghai Cancer Center, Shanghai, China; 208 Department of Pathology, GZA-ZNA Hospitals, Antwerp, Belgium; 209 Department of Radiation Oncology, Odette Cancer Centre, Sunnybrook Research Institute, Toronto, Canada; 210 Oncology Merck & Co, New Jersey, USA; 211 The Cancer Research Program, Garvan Institute of Medical Research, Darlinghurst, Australian Clinical Labs, Darlinghurst, Australia; 212 Georgetown University Medical Center, Washington DC, USA; 213 Department of Molecular and Experimental Medicine, Avera Cancer Institute, Sioux Falls, SD USA; 214 Translational Medicine, Bristol-Myers Squibb, Princeton, USA; 215 National Surgical Adjuvant Breast and Bowel Project Operations Center/NRG Oncology, Pittsburgh, USA; 216 Anatomic Pathology, Boston, MA USA; 217 King’s College London, London, UK; 218 Guy’s Hospital, London, UK; 219 Peking University First Hospital Breast Disease Center, Beijing, China; 220 Department of Pathology, Peter MacCallum Cancer Centre, Melbourne, Australia; 221 Dipartimento di Scienze della Salute (DSS), Firenze, Italy; 222 Department of Oncology, Champalimaud Clinical Centre, Lisbon, Portugal; 223 Department of Pathology and Laboratory Medicine, Tufts Medical Center, Boston, USA; 224 Department of Pathology, Fundación Valle del Lili, Cali, Colombia; 225 Department of Pathology, Montefiore Medical Center and the Albert Einstein College of Medicine, Bronx, NY USA; 226 Department of Pathology, University Hospital of Bellvitge, Oncobell, IDIBELL, L’Hospitalet del Llobregat, Barcelona, 08908 Catalonia Spain; 227 Department of Development and Regeneration, Laboratory of Experimental Urology, KU Leuven, Leuven, Belgium; 228 Department of Medical Oncology, Austin Health, Heidelberg, Australia; 229 Department of Surgery, Kansai Medical University to Tomoharu Sugie, Breast Surgery, Kansai Medical University Hospital, Hirakata, Japan; 230 Department of Pathology, Massachusetts General Hospital, Boston, USA; 231 Pathology Department, H.U. Vall d’Hebron, Barcelona, Spain; 232 Division of Bioinformatics and Biostatistics, U.S. Food and Drug Administration, Wuhan, USA; 233 Department of Pathology and Laboratory Medicine, Rhode Island Hospital and Lifespan Medical Center, Providence, USA; 234 Université Paris-Est, Créteil, France; 235 Praava Health, Dhaka, Bangladesh

**Keywords:** Prognostic markers, Tumour immunology, Breast cancer, Tumour biomarkers, Cancer imaging

## Abstract

Assessment of tumor-infiltrating lymphocytes (TILs) is increasingly recognized as an integral part of the prognostic workflow in triple-negative (TNBC) and HER2-positive breast cancer, as well as many other solid tumors. This recognition has come about thanks to standardized visual reporting guidelines, which helped to reduce inter-reader variability. Now, there are ripe opportunities to employ computational methods that extract spatio-morphologic predictive features, enabling computer-aided diagnostics. We detail the benefits of computational TILs assessment, the readiness of TILs scoring for computational assessment, and outline considerations for overcoming key barriers to clinical translation in this arena. Specifically, we discuss: 1. ensuring computational workflows closely capture visual guidelines and standards; 2. challenges and thoughts standards for assessment of algorithms including training, preanalytical, analytical, and clinical validation; 3. perspectives on how to realize the potential of machine learning models and to overcome the perceptual and practical limits of visual scoring.

## Introduction

Very large adjuvant trials have illustrated how the current schemes fail to stratify patients with sufficient granularity to permit optimal selection for clinical trials, likely owing to application of an overly limited set of clinico-pathologic features^1,2^. Histologic evaluation of tumor-infiltrating lymphocytes (TILs) is emerging as a promising biomarker in solid tumors and has reached level IB-evidence as a prognostic marker in triple-negative (TNBC) and HER2-positive breast cancer^3–5^. Recently, the St Gallen Breast Cancer Expert Committee endorsed routine assessment of TILs for TNBC patients^6^. In the absence of adequate standardization and training, visual TILs assessment (VTA) is subject to a marked degree of ambiguity and interobserver variability^7–9^. A series of published guidelines from this working group (also known as TIL Working group or TIL-WG) aimed to standardize VTA in solid tumors, to improve reproducibility and clinical adoption^10–12^. TIL-WG is an international coalition of pathologists, oncologists, statisticians, and data scientists that standardize the assessment of Immuno-Oncology Biomarkers to aid pathologists, clinicians, and researchers in their research and daily practice. The value of these guidelines was highlighted in two studies systematically examining VTA reproducibility^7,13^. Nevertheless, VTA continues to have inherent limitations that cannot be fully addressed through standardization and training, including: 1. visual assessment will always have some degree of inter-reader variability; 2. the time constraints of routine practice make comprehensive assessment of large tissue sections challenging^7,13^; 3. perceptual limitations may introduce bias in VTA, for example, the same TILs density is perceived to be higher if there is limited stroma.

Research in using machine learning (ML) algorithms to analyze histology has recently produced encouraging results, fueled by improvements in both hardware and methodology. Algorithms that learn patterns from labeled data, based on “deep learning” neural networks, have obtained promising results in many challenging problems. Their success has translated well to digital pathology, where they have demonstrated outstanding performance in tasks like mitosis detection, identification of metastases in lymph node sections, tissue segmentation, prognostication, and computational TILs assessment (CTA)^14–17^. ‘Traditional' computational analysis of histology focuses on complex image analysis routines, that typically require extraction of handcrafted features and that often do not generalize well across data sets^18,19^. Although studies utilizing deep learning-based methods suggest impressive diagnostic performance, and better generalization across data sets, these methods remain experimental. Table 1 shows a sample of published CTA algorithms and discusses their strengths and limitations, in complementarity with a previous literature review by the TIL-WG^16,20–31^.

**Table 1:** Sample CTA algorithms from the published literature.

Stain	Approach	Ref	Data set	Method	Ground truth	Notes
H&E	Patch classification	^24^	Multiple sites	CNN	Labeled patches (yes/no TILs)	Strengths: large-scale study with investigation of spatial TIL maps. AV includes molecular correlates.
			TCGA data set		Annotations are open-access	Limitations: does not distinguish sTIL and iTIL; does not classify individual TILs*.
						Other: we defined CTA TIL score as fraction of patches that contain TILs, and found this to be correlated with VTA (*R* = 0.659, *p* = 2e-35).
	Semantic segmentation	^16^	Breast	FCN	Traced region boundaries (exhaustive)	Strengths: large sample size and regions; investigates inter-rater variability at different experience levels; delineation of tumor, stroma and necrosis regions.
			TCGA data set		Annotations are open-access	Limitations: only detects dense TIL infiltrates*; does not classify individual TILs*.
	Semantic segmentation + Object detection	^25^	Breast	Seeding + FCN	Traced region boundaries (exhaustive)	Strengths: mostly follows TIL-WG VTA guidelines. AV includes correlation with consensus VTA scores and inter-pathologist variability.
			Private data set		Labeled & segmented nuclei within labeled region	Limitations: heavy ground truth requirement*; underpowered CV; and limited manually annotated slides.
	Object detection	^26^	Breast	SVM using morphology features	Labeled nuclei	Strengths: robust analysis and exploration of molecular TIL correlates.
			METABRIC data set		Qualitative density scores	Limitations: individual labeled nuclei are limited; does not distinguish TILs in different histologic regions*.
		^27^	Breast	RG and MRF	Labeled patches (low-medium-high density)	Strengths: explainable model and modular pipeline.
			Private data set			Limitations: does not distinguish sTIL and iTIL; does not classify individual TILs. Limited AV sample size.
		^28^	NSCLC	Watershed + SVM classifier	Labeled nuclei	Strengths: explainable model; robust CV; captures spatial TIL clustering.
			Private data sets			Limitations: limited AV; does not distinguish sTIL and iTIL.
	Object detection + inferred TIL localization	^31^	Breast	SVM classifier using morphology features	Labeled nuclei	Strengths: infers TIL localization using spatial localization. Robust CV. Investigation of spatial TIL patterns.
			METABRIC + private data sets		Qualitative density scores	Limitations: individual labeled nuclei are limited. not clear if spatial clustering has 1:1 correspondence with regions.
IHC	Object detection + manual regions	^29^	Colon	Complex pipeline (non-DL)	Overall density estimates	Strengths: CTA within manual regions, including invasive margin.
			Private data set			Limitations: unpublished AV.
	Object detection	^30^	Multiple	Multiple DL pipelines	Labeled nuclei within FOV (exhaustive)	Strengths: large-scale, robust AV. Systematic benchmarking.
			Private data set			Limitations: no CV; does not distinguish TILs in different regions*.

**Fig. 1:**
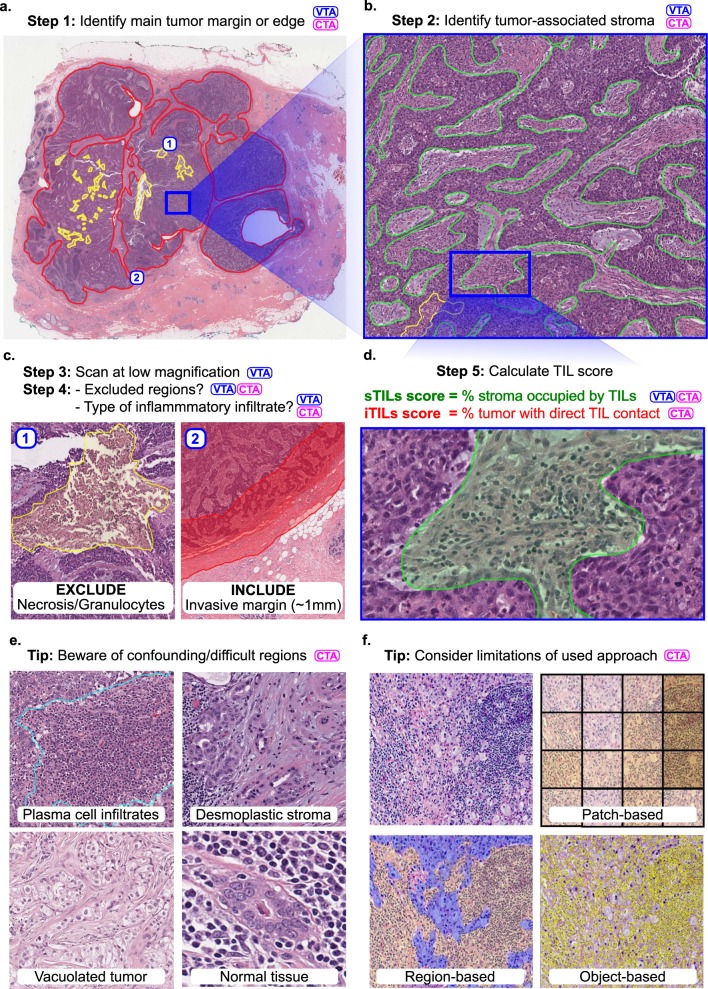
Outline of the visual (VTA) and computational (CTA) procedure for scoring TILs in breast carcinomas. TIL scoring is a complex procedure, and breast carcinomas are used as an example. Specific guidelines for scoring different tumors are provided in the references. Steps involved in VTA and/or CTA are tagged with these abbreviations. CTA according to TIL-WG guidelines involves TIL scoring in different tissue compartments. **a** Invasive edge is determined (red) and key confounding regions like necrosis (yellow) are delineated. **b** Within the central tumor, tumor-associated stroma is determined (green). Other considerations and steps are involved depending on histologic subtype, slide quality, and clinical context. **c** Determination of regions for inclusion or exclusion in the analysis in accordance with published guidelines. **d** Final score is estimated (visually) or calculated (computationally). In breast carcinomas, stromal TIL score (sTIL) is used clinically. Intratumoral TIL score (iTIL) is subject to more VTA variability, which has hampered the generation of evidence demonstrating prognostic value; perhaps CTA of iTILs will prove less variable and, consequently, prognostic. **e** The necessity of diverse pathologist annotations for robust analytical validation of computational models. Desmoplastic stroma may be misclassified as tumor regions; Vacuolated tumor may be misclassified as stroma; intermixed normal acini or ducts, DCIS/LCIS, and blood vessels may be misclassified as tumor; plasma cells are sometimes misclassified as carcinoma cells. Note that while the term “TILs” includes lymphocytes, plasma cells and other small mononuclear infiltrates, lumping these categories may not be optimal from an algorithm design perspective; plasma cells tend to be morphologically different from lymphocytes in nuclear texture, size, and visible cytoplasm. **f** Various computational approaches may be used for computational scoring. The more granular the algorithm is, the more accurate/useful it is likely to be, but—as a trade-off—the more it relies on exhaustive manual annotations from pathologists. The least granular approach is patch classification, followed by region delineation (segmentation), then object detection (individual TILs). A robust computational scoring algorithm likely utilizes a combination of these (and related) approaches.

This non-exhaustive list has been restricted to H&E and chromogenic IHC, although excellent works exist showing CTA based on other approaches like multiplexed immunofluorescence^21–23^. Published CTA algorithms vary markedly in their approach to TIL scoring, the robustness of their validation, their interpretability, and their consistency with published VTA guidelines. Strengths and limitations of each publication is highlighted, with general limitations (related to the broad approach used, not the specific paper) are marked with an asterisk (*). Going forward, nuanced approaches are needed, ideally incorporating workflows for robust quantification and validation as presented in this paper. Different approaches have different ground truth requirements (illustrated in Fig. 1, panel f), hence the need for large-scale ground truth data sets. We encourage all future CTA publications to open-access their data sets whenever possible. Of note are two major efforts: 1. A group of scientists, including the US FDA and the TIL-WG, is collaborating to crowdsource pathologists and collect images and pathologist annotations that can be qualified by the FDA medical device development tool program; 2. The TIL-WG is organizing a challenge to validate CTA algorithms against clinical trial outcome data (CV).

*AV* analytical validation, *CNN* convolutional neural network, *DL* deep learning, *FCN* fully convolutional network, *FOV* field of view, *MRF* markov random field, *RG* region growing, *NSCLC* non-small cell lung cancer, *SVM* support vector machine.

This review and perspective provides a broad outline of key issues that impact the development and translation of computational tools for TILs assessment. The ideal intended outcome is that CTA is successfully integrated into the routine clinical workflow; there is significant potential for CTA to address inherent limitations in VTA, and partially to mitigate high clinical demands in remote and under-resourced settings. This is not too difficult to conceive, and there are documented success stories in the commercialization and clinical adoption of computational algorithms including pap smear cytology analyzers^32^, blood analyzers^33^, and automated immunohistochemistry (IHC) workflows for ER, PR, Her2, and Ki67^34–38^.

## The impact of staining approach on algorithm design and deployment

The type of stain and imaging modality will have a significant impact on algorithm design, validation, and capabilities. VTA guideline from the TIL-WG focus on assessment of stromal TILs (sTIL) using hematoxylin and eosin (H&E)-stained formalin-fixed paraffin-embedded sections, given their practicality and widespread availability, and the clear presentation of tissue architecture this stain provides^10–12,39^. Multiple studies have relied on in situ approaches like IHC, in situ hybridization (ISH), or genomic deconvolution in assessing TILs^11,40,41^. These modalities, however, are not typically used in daily clinical TILs assessment, as they are either still experimental, rely on assays of variable reliability, or involve stains not widely used in clinical practice, especially in low-income settings^4,10,11^. It is also difficult to quantitate and establish consistent thresholds for IHC measurement of even well-defined epitopes, such as Ki67 and ER, between different labs^42,43^. Moreover, there is no single IHC stain that highlights all mononuclear cells with high sensitivity and specificity, so H&E remains the stain typically used in the routine clinical setting^44^.

Despite these issues, there are significant potential advantages for using IHC with CTAs. By specifically staining TILs, IHC can make image analysis more reliable, and can also present new opportunities for granular TILs subclassification; different TIL subpopulations, including CD4+ T cells, CD8+ T cells, Tregs, NK cells, B cells, etc, convey pertinent information on immune activation and repression^4,12^. IHC is already utilized in standardization efforts for TILs assessment in colorectal carcinomas^45,46^. The specific highlighting of TILs by IHC can improve algorithm specificity^47,48^, and enable characterization of TIL subpopulations that have potentially distinct prognostic or predictive roles^49,50^. IHC can reduce misclassification of intratumoral TILs, which are difficult to reliably assess given their resemblance to tumor or supporting cells in many contexts like lobular breast carcinomas, small-blue-cell tumors like small cell lung cancer, and primary brain tumors^4,12^.

## Characteristics of CTA algorithms that capture clinical guidelines

TIL-WG guidelines for VTA are somewhat complex^4,10–12^. There are VTA guidelines for many primary solid tumors and metastatic tumor deposits^10,12^, for untreated infiltrating breast carcinomas^11^, post-neoadjuvant residual carcinomas of the breast^39^, and for carcinoma in situ of the breast^39^. TILs score is defined as the fraction of a tissue compartment that is occupied by TILs (lymphoplasmacytic infiltrates). Different compartments have different prognostic relevance; tumor-associated sTILs is the most relevant in most solid tumors, whereas intratumoral TIL score (iTILs) has been reported to be prognostic, most notably in melanoma^10^. The spatial and visual context of TILs is strongly confounded by organ site, histologic subtype, and histomorphologic variables; therefore, it is important to provide situational context and instructions for clinical use of the CTA algorithms^24,51,52^. For example, a CTA algorithm designed for general-purpose breast cancer TILs scoring should be validated on different subtypes (infiltrating ductal, infiltrating lobular, mucinous, etc) and on a wide array of slides that capture variabilities in tumor phenotype (e.g., vacuolated tumor, necrotic tumor, etc), stromal phenotype (e.g., desmoplastic stroma), TIL densities, and sources of variability like staining and artifacts. That being said, it is plausible to assume that the biology and significance of TILs may vary in different clinical and genomic subtypes of the same primary cancer site, and that a general-purpose TILs-scoring algorithm may not be applicable. Further research into the commonalities and differences in the prognostic and biological value of TILs in different tissue sites and within different subtypes of the same cancer is warranted.

Clear inclusion criteria are helpful in deciding whether a slide is suitable for a particular CTA algorithm. For robust implementation, it is useful to: 1. detect when slides fail to meet its minimum quality; 2. provide some measure of confidence in its predictions; 3. be free of single points of failure (i.e., modular enough to tolerate failure of some sub-components); 4. be somewhat explainable, such that an expert pathologist can understand its limitations, common failure modes, and what the model seems to rely on in making decisions. Algorithms for measuring image quality and detecting artifacts will play an important role in the clinical implementation of CTA^53^.

From a computer vision perspective, we can subdivide CTA in two separate tasks: 1. segmentation of the region of interest (e.g., intratumoral stroma in case of sTIL assessment) and 2. detection of individual TILs within that region. In practice, a set of complementary computer vision problems often need to be addressed to score TILs (Fig. 1). To segment the region in which TILs will be assessed, it is also often needed to explicitly segment regions for exclusion from the analysis. Although these can be manually annotated by pathologists, these judgements are a significant source of variability in VTA, and developing algorithms capable of performing these tasks could improve reproducibility and standardization^7–9^.

Specifically, segmentation of the “central tumor” and the “invasive margin/edge” enable TILs quantitation to be focused in relevant areas, excluding “distant” stroma along with normal tissue and surrounding structures. A semi-precise segmentation of invasive margin also allows sTILs score to be broken down for the margin and central tumor regions (especially, in colorectal carcinomas) and to characterize peri-tumoral TILs independently^10^. Within the central tumor, segmenting carcinoma cell nests and intratumoral stroma enables separate measurements for sTIL and iTIL densities. Furthermore, segmentation helps exclude key confounder regions that need to be excluded from the analysis. This includes necrosis, tertiary lymphoid structures, intermixed normal tissue or DCIS/LCIS (in breast carcinoma), pre-existing lymphoid stroma (in lymph nodes and oropharyngeal tumors), perivascular regions, intra-alveolar regions (in lung), artifacts, etc. This step requires high-quality segmentation annotations, and may prove to be challenging. Indeed, for routine clinical practice, it may be necessary to have a pathologist perform a quick visual confirmation of algorithmic region segmentations, and/or create high-level region annotations that may be difficult to produce algorithmically.

When designing a TIL classifier, consideration of key confounding cells is important. Although lymphocytes are, compared with tumor cells, relatively monomorphic, their small sizes offer little lymphocyte-specific texture information; small or perpendicularly cut stromal cells and even prominent nucleoli may result in misclassifications. Apoptotic bodies, necrotic debris, neutrophils, and some tumor cells (especially in lobular breast carcinomas and small-blue-round cell tumors) are other common confounders. Quantitation of systematic misclassification errors is warranted; some misclassifications will have contradictory consequences for clinical decision making. For example, neutrophils are evidently associated with adverse clinical outcomes, whereas TILs are typically associated with favorable outcomes^51^. Note that some of the TIL-WG clinical guidelines have been optimized for human scoring and are not very applicable in CTA algorithm design. For example, in breast carcinomas it is advised to “include but not focus on” tumor invasive edge TILs and TILs “hotspots”; CTA circumvents the need to address these cognitive biases^11^. To fully adhere to clinical guidelines, segmentation of TILs is warranted, so that the fraction of intratumoral stroma occupied by TILs is calculated.

## Computer-aided versus fully automated TILs assessment

The extent to which computational tools can be used to complement clinical decision making is highly context-dependent, and is strongly impacted by cancer type and clinical setting^54–57^. In a computer-aided diagnosis paradigm, CTA is only used to provide guidance and increase efficiency in the workflow by any combination of the following: 1. calculating overall TILs score estimates to provide a frame-of-reference for the visual estimate; 2. directing the pathologist attention to regions of interest for TIL scoring, helping mitigate inconsistencies caused by heterogeneity in TILs density in different regions within the same slide; 3. providing a quantitative estimate for TILs density within regions of interest that the pathologist identifies, hence reducing ambiguity in visual estimation. Two models exist to assess this type of workflow during model development. In the traditional open assessment framework, the algorithm is trained on a set of manually annotated data points and evaluated on an independent held-out testing set. Alternatively, a closed-loop framework may be adopted, whereby pathologists can use the algorithmic output to re-evaluate their original decisions on the held-out set after exposure to the algorithmic results^55,56^. Both frameworks have pros and cons, although the closed-loop framework enables assessment of the potential impact that CTA has on altering the clinical decision-making process^56^.

**Fig. 2:**
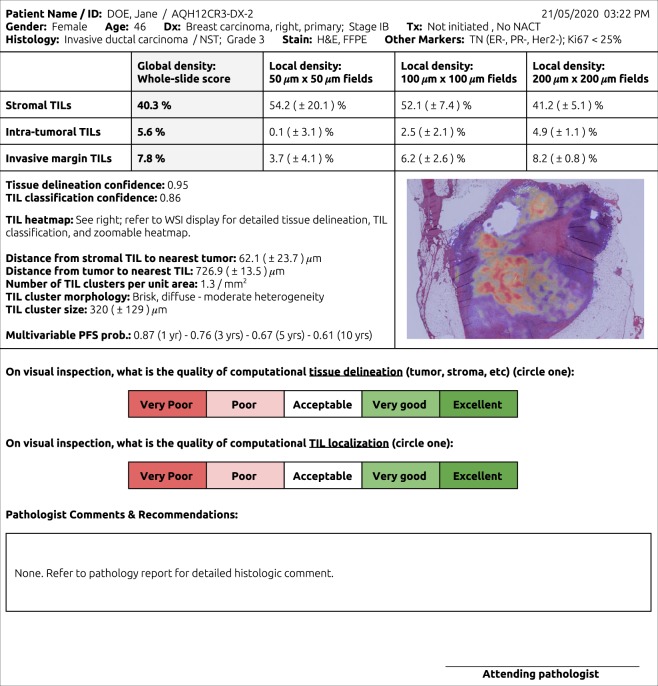
Conceptual pathology report for computational TIL assessment (CTA). CTA reports might include global TIL estimates, broken down by key histologic regions, and estimates of classifier confidence. CTA reports are inseparably linked to WSI viewing systems, where algorithmic segmentations and localizations supporting the calculated scores are displayed for sanity check verification by the attending pathologist. Other elements, like local TIL estimates, TIL clustering results, and survival predictions may also be included.

The alternative paradigm is an entirely computational pipeline for CTA. This approach clearly provides efficiency gains, which could markedly reduce costs and accelerate development in a research setting. When the sample sizes are large enough, a few failures (i.e., “noise”) could be tolerated without altering the overall conclusions. This is contrary to clinical medicine, where CTA is expected to be highly dependable for each patient, especially when it is used to guide treatment decisions. Owing to the highly consequential nature of medical decision-making, a stand-alone CTA algorithm requires a higher bar for validation. It is also likely that even validated stand-alone CTA tools will need “sanity checks” by pathologists, guarding against unexpected failures. For example, a CTA report may be linked to a WSI display system to visualize the intermediate results (i.e., detected tissue boundaries and TILs locations) that were used by the algorithm to reach its decision (Fig. 2).

We do not envision computational models at their current level of performance replacing pathologist expertize. In fact, we would argue that quite the opposite is true; CTA enables objective quantitative assessment of an otherwise ambiguous metric, enabling the pathologist to focus more of his/her time on higher-order decision-making tasks^54^. With that in mind, we argue that the efficiency gains from CTA in under-resourced settings are likely to be derived from workflow efficiency, as opposed to reducing the domain expertize required to make diagnostic and therapeutic assessments. When used in a telepathology setting, i.e., off-site review of WSIs, CTA is still likely to require supervision by an experienced attending pathologist. Naturally, this depends on infrastructure, and one may argue that the cost-effectiveness of CTA is determined by the balance between infrastructure costs (WSI scanners, computing facilities, software, cloud support, etc) and expected long-term efficiency gains.

## Validation and training issues surrounding computational TIL scoring

CTA algorithms will need to be validated just like any prognostic or predictive biomarker to demonstrate preanalytical validation (Pre-AV), analytical validation (AV), clinical validation (CV), and clinical utility^8,58,59^. In brief, Pre-AV is concerned with procedures that occur before CTA algorithms are applied, and include items like specimen preparation, slide quality, WSI scanner magnification and specifications, image format, etc; AV refers to accuracy and reproducibility; CV refers to stratification of patients into clinically meaningful subgroups; clinical utility refers to overall benefit in the clinical setting, considering existing methods and practices. Other considerations include cost-effectiveness, implementation feasibility, and ethical implications^59^. VTA has been subject to extensive AV, CV, and clinical utility assessment, and it is critical that CTA algorithms are validated using the same high standards^7,8^. The use-case of a CTA algorithm, specifically whether it is used for computer-aided assessment or for largely unsupervised assessment, is a key determinant of the extent of required validation. Key resources to consult include: 1. Recommendations by the Society for Immunotherapy of Cancer, for validation of diagnostic biomarkers; 2. Guidance documents by the US Food and Drug Administration (FDA); 3. Guidelines from the College of American Pathologists, for validation of diagnostic WSI systems^60–64^. Granted, some of these require modifications in the CTA context, and we will highlight some of these differences here.

Pre-AV is of paramount importance, as CTA algorithm performance may vary in the presence of artifacts, variability in staining, tissue thickness, cutting angle, imaging, and storage^65–68^. Trained pathologists, on the other hand, are more agile in adapting to variations in tissue processing, although these factors can still impact their visual assessment. Some studies have shown that the implementation of a DICOM standard for pathology images can improve standardization and improve interoperability if adopted by manufacturers^67,69^. Techniques for making algorithms robust to variations, rather than eliminating the variations, have also been widely studied and are commonly employed^69–72^. According to CAP guidelines, it is necessary to perform in-house validation of CTAs in all pathology laboratories, to validate the entire workflow (i.e., for each combination of tissue, stain, scanner, and CTA) using adequate sample size representing the entire diagnostic spectrum, and to re-validate whenever a significant component of the pre-analytic workflow changes^62^. Pre-AV and AV are most suitable in the in-house validation setting, as they can be performed with relatively fewer slides. It may be argued that proper in-house Pre-AV and AV suffice, provided large-scale prospective (or retrospective-prospective) AV, CV, and Clinical Utility studies were performed in a multi-center setting. Demonstrating local equivalency of Pre-AV and AV results can thus allow “linkage” to existing CV and Clinical Utility results assuming comparable patient populations.

AV typically involves quantitative assessment of CTA algorithm performance using ML metrics like segmentation or classification accuracy, prediction vs truth error, and area under receiver–operator characteristic curve or precision-recall curves. AV also includes validation against “non-classical” forms of ground truth like co-registered IHC, in which case the registration process itself may also require validation. AV is a necessary prerequisite to CV as it answers the more fundamental question: “Do CTA algorithms detect TILs correctly?”. AV should measure performance over the spectrum of variability induced by pre-analytic factors, and in cohorts that reflect the full range of intrinsic/biological variability. Naturally, this means that uncommon or rare subtypes of patterns are harder to validate owing to sample size limitations. AV of nucleus detection and classification algorithms has often neglected these issues, focusing on a large number of cells from a small number of cases.

Demonstrating the validity and generalization of prediction models is a complex process. Typically, the initial focus is on “internal” validation, using techniques like split-sample cross validation and bootstrapping. Later, the focus shifts to “external” validation, i.e., on an independent cohort from another institution. A hybrid technique called “internal–external” (cross-) validation may be appropriate when multi-institutional data sets (like the TCGA and METABRIC) are available, where training is performed on some hospitals/institutions and validation is performed on others. This was recommended by Steyerberg and Harrell and used in some computational pathology studies^16,73–75^.

Many of the events associated with cancer progression and subtyping are strongly correlated, so it may not be enough to show correspondence between global/slide-level CTA and VTA scores, as this shortcuts the AV process^49^. AV therefore relies on the presence of quality “ground truth” annotations. Unfortunately, there is a lack of open-access, large-scale, multi-institutional histology segmentation and/or TIL classification data sets, with few exceptions^16,24,76,77^. To help address this, a group of scientists, including the US FDA Center for Devices and Radiological Health (CDRH) and the TIL-WG, is collaborating to crowdsource pathologists and collect images and pathologist annotations that can be qualified by the FDA/CDRH medical device development tool program (MDDT). The MDDT qualified data would be available to any algorithm developer to be used for the analytic evaluation of their algorithm performance in a submission to the FDA/CDRH^78^. The concept of “ground truth” in pathology can be vague and is often subjective, especially when dealing with H&E; it is therefore important to measure inter-rater variability by having multiple experts annotate the same regions and objects^7,8^. A key bottleneck in this process is the time commitment of pathologists, so collaborative, educational and/or crowdsourcing settings can help circumvent this limitation^16,79^. It should be stressed, however, that although annotations from non-pathologists or residents may be adequate for CTA algorithm training; validation may require ground truth annotations created or reviewed by experienced practicing pathologists^16,80^.

It is important to note that the ambiguity in ground truth (even if determined by consensus by multiple pathologists) typically warrants additional validation using objective criteria, most notably the ability to predict concrete clinical endpoints in validated data sets. One of the best ways to meet this validation bar is to use WSIs from large, multi-institutional randomized-controlled trials. To facilitate this effort, the TIL-WG is establishing strategic international partnerships to organize a machine learning challenge to validate CTA algorithms using clinical trials data. The training sets would be made available for investigators to train and fine tune their models, whereas separate blinded validation sets would only be provided once a locked-down algorithm has been established. Such resources are needed so that different algorithms and approaches can be directly compared on the same, high-quality data sets.

## CTA for clinical versus academic use

Like VTA, CTA may be considered to fall under the umbrella of “imaging biomarkers,” and likely follows a similar validation roadmap to enable clinical translation and adoption^38,81,82^. CTA may be used in the following academic settings, to name a few: 1. as a surrogate marker of response to experimental therapy in animal models; 2. as a diagnostic or predictive biomarker in retrospective clinical studies using archival WSI data; 3. as a diagnostic or predictive biomarker in prospective randomized-controlled trials. Incorporation of imaging biomarkers into prospective clinical trials requires some form of analytical and clinical validation (using retrospective data, for example), resulting in the establishment of Standards of Practice for trial use^81^. Establishment of clinical validity and utility in multicentric prospective trials is typically a prerequisite for use in day-to-day clinical practice. In a research environment, it is not unusual for computational algorithms to be frequently tweaked in a closed-loop fashion. This tweaking can be as simple as altering hyper-parameters, but can include more drastic changes like modifications to the algorithm or (inter)active machine learning^83,84^. From a standard regulatory perspective, this is problematic as validation requires a defined “lockdown” and version control; any change generally requires at least partial re-validation^64,85^. It is therefore clear that the most pronounced difference between CTA use in basic/retrospective research, prospective trials, and routine clinical setting is the rigor of validation required^38,81,82^.

In a basic/retrospective research environment, there is naturally a higher degree of flexibility in adopting CTA algorithms. For example, all slides may be scanned using the same scanner and using similar tissue processing protocols. In this setting, there is no immediate need for worrying about algorithm generalization performance under external processing or scanning conditions. Likewise, it may not be necessary to validate the model using ground truth from multiple pathologists, especially if some degree of noise can be tolerated. Operational issues and practicality also play a smaller role in basic/retrospective research settings; algorithm speed and user friendliness of a particular CTA algorithm may not be relevant when routine/repetitive TILs assessment is not needed. Even the nature of CTA algorithms may be different in a non-clinical setting. For instance, even though there is conflicting evidence on the prognostic value of iTILs in breast cancer, there are motivations to quantify them in a research environment. It should be noted, however, that this flexibility is only applicable for CTA algorithms that are being used to support non-clinical research projects, not for those algorithms that are being validated for future clinical use.

## The future of computational image-based immune biomarkers

CTA algorithms can enable characterization of the tumor microenvironment beyond the limits of human observers, and will be an important tool in identifying latent prognostic and predictive patterns of immune response. For one, CTA enables calculation of local TIL densities at various scales, which may serve as a guide to “pockets” of differential immune activation (Fig. 2). This surpasses what is possible with VTA and such measurements are easy to calculate provided that CTA algorithms detect TILs with adequate sensitivity and specificity. Several studies have identified genomic features that in hindsight are associated with TILs, and CTA presents opportunities for systematic investigation of these associations^24,26,74,86,87^. The emergence of assays and imaging platforms for multiplexed immunofluorescence and in situ hybridization will present new horizons for identifying predictive immunologic patterns and for understanding the molecular basis of tumor-immune interactions^88,89^; these approaches are increasingly becoming commoditized.

Previous work examined how various spatial metrics from cancer-associated stroma relate to clinical outcomes, and similar concepts can be borrowed; for example, metrics capturing the complex relationships between TILs and other cells/structures in the tumor microenvironment^90^. CTA may enable precise definitions of “intratumoral stroma”, for example using a quantitative threshold (i.e., “stroma within x microns from nearest tumor nest”). Similar concepts could be applied when differentiating tertiary lymphocytic aggregates, or other TIL hotspots, from infiltrating TILs that presumably have a direct role in anticancer response. It is also important to note that lymphocytic aggregation and other higher-order quantitative spatial metrics may play important prognostic roles yet to be discovered. A CTA study identified five broad categories of spatial organization of TILs infiltration, which are differentially associated with different cancer sites and subtypes^24^. Alternatively, TILs can be placed on a continuum, such that sTILs that have a closer proximity to carcinoma nests get a higher weight. iTILs could be characterized using similar reasoning. Depending on available ground truth, numerous spatial metrics can be calculated. Nuanced assessment of immune response can be performed; for example, number of apoptotic bodies and their relation to nearby immune infiltrates. It is likely that there would be a considerable degree of redundancy in the prognostic value of CTA metrics; such redundancy is not uncommon in genomic biomarkers^91^. This should not be problematic as long as statistical models properly account for correlated predictors. In fact, the ability to calculate numerous metrics for a very large volume of cases enables large-scale, systematic discovery of histological biomarkers, bringing us a step closer to evidence-based pathology practice.

Learning-based algorithms can be utilized to learn prognostic features directly from images in a minimally biased manner (without explicit detection of TILs), and to integrate these with standard clinico-pathologic and genomic predictors. The approach of using deep learning algorithms to first detect and classify TILs and structures in histology, and then to calculate quantitative features of these objects, presents a way of closely modeling the clinical guidelines set forth by expert pathologists. Here, the power of learning algorithms is directed at providing highly accurate and robust detection and classification to enable reproducible and quantitative measurement. Although this approach is interpretable and provides a clear path for analytic validation, the limitation is that quantitative features are prescribed instead of learned. Recently, there have been successful efforts to develop end-to-end prognostic deep learning models that learn to directly predict clinical outcomes from raw images without any intermediate classification of histologic objects like TILs^17,92^. Although these end-to-end learning approaches have the potential to learn latent prognostic patterns (including those impossible to assess visually), they are less interpretable and thus the factors driving the predictions are currently unknown.

Finally, we would note that one of the key limitations of machine learning models, and deep learning models in particular, is their opaqueness. It is often the case that model accuracy comes at a cost to explainability, giving rise to the term “black box” often associated with deep learning. The problem with less explainable models is that key features driving output may not be readily identifiable to evaluate biologic plausibility, and hence the only safeguard against major flaws is extensive validation^93^. Perhaps the most notorious consequence of this problem is “adversarial examples”, which are images that look natural to the human eye but that are specifically crafted (e.g., by malicious actors) to mislead deep learning models to make targeted misclassifications^94^. Nevertheless, recent advances in deep learning research have substantially increased model interpretability, and have devised key model training strategies (e.g., generative adversarial neural networks) to increase performance robustness^93,95–97^.

## Conclusions

Advances in digital pathology and ML methodology have yielded expert-level performance in challenging diagnostic tasks. Evaluation of TILs in solid tumors is a highly suitable application for computational and computer-aided assessment, as it is both technically feasible and fills an unmet clinical need for objective and reproducible assessment. CTA algorithms need to account for the complexity involved in TIL-scoring procedures, and to closely follow guidelines for visual assessment where appropriate. TIL scoring needs to capture the concepts of stromal and intratumoral TILs and to account for confounding morphologies specific to different tumor sites, subtypes, and histologic patterns. Preanalytical factors related to imaging modality, staining procedure, and slide inclusion criteria are critical considerations, and robust analytical and clinical validation is key to adoption. In the clinical setting, CTA would ideally provide time- and cost-savings for pathologists, who face increasing demands for reporting biomarkers that are time-consuming to evaluate and subject to considerable inter- and intra- reader variability. In addition, CTA enables discovery of complex spatial patterns and genomic associations beyond the limits of visual scoring, and presents opportunities for precision medicine and scientific discovery.
